# (3a*R*,6*S*,7a*R*)-7a-Chloro-2-[(4-nitro­phen­yl)sulfon­yl]-1,2,3,6,7,7a-hexa­hydro-3a,6-ep­oxy­iso­indole

**DOI:** 10.1107/S1600536813025336

**Published:** 2013-09-18

**Authors:** Ersin Temel, Aydın Demircan, Muhammet Kasım Kandemir, Medine Çolak, Orhan Büyükgüngör

**Affiliations:** a Ondokuz Mayıs University, Arts and Sciences Faculty, Department of Physics, TR-55139 Samsun, Turkey; b Department of Chemistry, Faculty of Arts and Sciences, Niĝde University, TR-51240 Niĝde, Turkey

## Abstract

In the title compound, C_14_H_13_ClN_2_O_5_S, the chlorine-substituted tetrahydrofuran ring adopts a twist conformation and the other tetra­hydro­furan ring an envelope conformation with the O atom as the flap. The pyrrolidine ring adopts a twist conformation. In the crystal, C—H⋯O hydrogen bonds link the mol­ecules into zigzag chains running along the *b-*axis direction.

## Related literature

For Diels–Alder reactions, see: Winkler (1996 ▶); Paulvannan (2004 ▶); Norton (1942 ▶); Fraile *et al.* (2001 ▶); Padwa *et al.* (2003 ▶); Medimagh *et al.* (2008 ▶); Avalos *et al.* (2003 ▶). For the thermal IMDA reaction of furan-cored compounds, see: Karaarslan & Demircan (2006 ▶); Koşar *et al.* (2006 ▶, 2007 ▶, 2011 ▶); Arslan *et al.* (2008 ▶); Temel *et al.* (2012 ▶). For graph-set notation of hydrogen bonds, see: Bernstein *et al.* (1995 ▶). For puckering parameters, see: Cremer & Pople (1975 ▶).
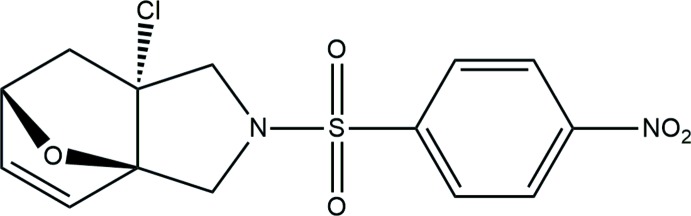


## Experimental

### 

#### Crystal data

C_14_H_13_ClN_2_O_5_S
*M* *_r_* = 356.77
Monoclinic, 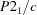

*a* = 7.5193 (3) Å
*b* = 9.7278 (4) Å
*c* = 20.7616 (7) Å
β = 93.659 (3)°
*V* = 1515.54 (10) Å^3^
*Z* = 4
Mo *K*α radiation
μ = 0.42 mm^−1^
*T* = 296 K
0.68 × 0.63 × 0.60 mm

#### Data collection

STOE IPDS 2 diffractometer
Absorption correction: integration (*X-RED32*; Stoe & Cie, 2002 ▶) *T*_min_ = 0.811, *T*_max_ = 0.850
11192 measured reflections
3151 independent reflections
2494 reflections with *I* > 2σ(*I*)
*R*_int_ = 0.242

#### Refinement

*R*[*F* ^2^ > 2σ(*F* ^2^)] = 0.036
*wR*(*F* ^2^) = 0.110
*S* = 1.03
3151 reflections
208 parameters
H-atom parameters constrained
Δρ_max_ = 0.26 e Å^−3^
Δρ_min_ = −0.23 e Å^−3^

### 

Data collection: *X-AREA* (Stoe & Cie, 2002 ▶); cell refinement: *X-AREA*; data reduction: *X-RED32* (Stoe & Cie, 2002 ▶); program(s) used to solve structure: *SHELXS97* (Sheldrick, 2008 ▶); program(s) used to refine structure: *SHELXL97* (Sheldrick, 2008 ▶); molecular graphics: *ORTEP-3 for Windows* (Farrugia, 1997 ▶) and *Mercury* (Macrae *et al.*, 2006 ▶); software used to prepare material for publication: *WinGX* (Farrugia, 1999 ▶).

## Supplementary Material

Crystal structure: contains datablock(s) I, global. DOI: 10.1107/S1600536813025336/bt6932sup1.cif

Structure factors: contains datablock(s) I. DOI: 10.1107/S1600536813025336/bt6932Isup2.hkl

Supplementary material file. DOI: 10.1107/S1600536813025336/bt6932Isup3.cml

Additional supplementary materials:  crystallographic information; 3D view; checkCIF report

## Figures and Tables

**Table 1:** Hydrogen-bond geometry (Å, °)

*D*—H⋯*A*	*D*—H	H⋯*A*	*D*⋯*A*	*D*—H⋯*A*
C2—H2⋯O4^i^	0.93	2.58	3.437 (3)	154
C14—H14*B*⋯O5^ii^	0.97	2.54	3.317 (2)	137

Symmetry codes: (i) 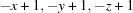; (ii) 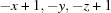.

**Fig. 1.**
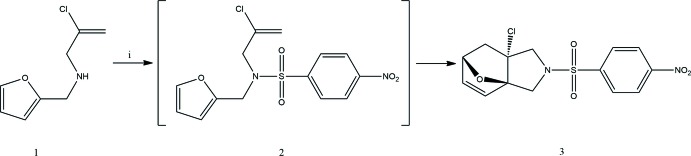
Synthesis of the title compound.

**Fig. 2.**
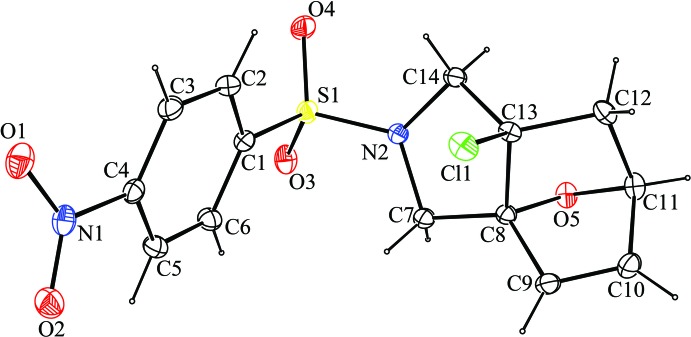
The molecular structure of the title compound, with displacement ellipsoids drawn at the 30% probability level.

**Fig. 3.**
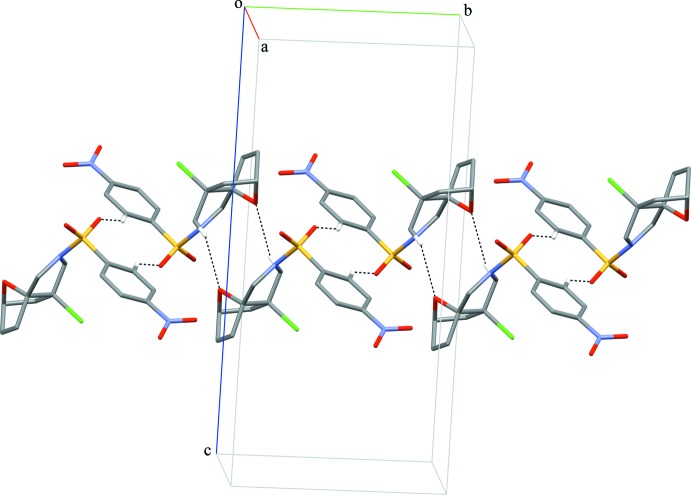
Part of the crystal structure of the title compound, showing the formation of R22(10) rings.

## supplementary crystallographic information

### 1. Comment

The Diels Alder reaction is one of the most powerful and useful cycloaddition reactions in synthetic organic chemistry (Winkler, 1996; Paulvannan, 2004; Norton, 1942). Intra-molecular Diels Alder (IMDA) reaction should be taken into account for synthesis of a molecule containing a six membered ring fused to a second ring. The feasibility of employing IMDA has been considered the main application often becomes the preparation of the substrates in synthetic pathways (Fraile *et al.*, 2001; Padwa *et al.*, 2003). Thermal intramolecular Diels Alder reaction has also been popular and facile methodology since early 1980's Furan is also one of the most used diene part in thermal IMDA cycloaddition (Medimagh *et al.*, 2008; Avalos *et al.*, 2003).

We have been working on thermal IMDA reaction of furan cored compounds in which side chain of furan includes oxygen, sulfur and nitrogen (Karaarslan *et al.*, 2006; Koşar *et al.*, 2006, 2007, 2011; Arslan *et al.*, 2008; Temel *et al.*, 2012). Here, we report that the new isoxazol cycloadduct, 3 in aqueous condition is furnished in one pot reaction from seconder amine, 1. We assume that the protective group on nitrogen; *p*-Nosyl behaves as steric buttress and accelerates the cycloaddition progress right after the protection stage, 2. The improvement of the technology and economic reaction are achieved, two processes; protection and IMDA cycloaddition stages are performed in one pot process. We consider that the improvement of this methodology will possibly allow organic chemist to a facile access to important classes of azaheterocycles (Figure 1).

The title compound contains epoxyisoindole and phenyly rings linked through N—S—C bridge in the unit cell (Fig. 2). Epoxyisoindole moiety is formed with fused a six-membered ring and three five-membered rings which are puckered. Of the tetrahydrofuran rings, the one which is attached to chloride, O_5_/C_11–13_/C_8_, adopts a half-chair conformation while the other one, O_5_/C_8–11_, adopts an envelope conformation with the puckering parameters of Q=0.6011 (18) Å, 0.5037 (19) Å and φ=1.95 (19)°, 180.8 (3)°, respectively. The five-membered pyrrolidine ring twisted on C_8_—C_13_ atoms deviated from the mean plane by about -0.1804 (11) Å and 0.1969 (11) Å, respectively, with the puckering parameters of Q=0.3177 (18) Å and φ=278.7 (3)°. The six-membered ring, C_8–13_, has a boat conformation, according to the puckering parameters [Q=0.948 (2) Å, θ=89.29 (12)° and φ=180.53 (13)°] (Cremer & Pople, 1975).

The crystal packing of (I) is provided by inter-molecular C2—H2—O4 and C14—H14B···O5 hydrogen bonds which are generate dimeric *R*^2^_2_(10) rings running parallel to the *b* axis (Bernstein *et al.*, 1995) (Fig. 3).

### 2. Experimental

2-chloro-*N*-(furan-2-ylmethyl)prop-2-en-1-amine, 1 (0.92 g, 5.38 mmol) in water (50 ml) was added *p*-nitrobenzenesulfonyl chloride (1.43 g, 6.45 mmol) portion wise followed by potassium carbonate (0.9 g, 6.45 mol). The reaction mixture was stirred for 48 h at 396 K. The reaction mixture was allowed to room temperature and added NaOH 10% (35 ml). The mixture was then extracted with ethyl acetate (3x35 ml) and brine (35 ml). Combined organic phases was dried over magnesium sulfate, filtered and evaporated. The purification by column chromatography afforded colourless crystals (1.17 g, 61% yield). Recrystallization was performed in DCM:Hexane. t.l.c., (Hexane:Ethylacetate); (7:3), Rf; 0.49. Melting Point: 421–423 K.

### 3. Refinement

H atoms were positioned geometrically and treated using a riding model, fixing the bond lengths at 0.97, 0.98 and 0.93 Å for CH_2_, CH and CH(aromatic), respectively. The displacement parameters of the H atoms were constrained with *U*_iso_(H) = 1.2*U*_eq_ (aromatic, methylene or methine C).

### Crystal data

**Table:**

C_14_H_13_ClN_2_O_5_S	*F*(000) = 736
*M**_r_* = 356.77	*D*_x_ = 1.564 Mg m^−^^3^
Monoclinic, *P*2_1_/*c*	Mo *K*α radiation, λ = 0.71073 Å
Hall symbol: -P 2ybc	Cell parameters from 11192 reflections
*a* = 7.5193 (3) Å	θ = 2.0–28.0°
*b* = 9.7278 (4) Å	µ = 0.42 mm^−^^1^
*c* = 20.7616 (7) Å	*T* = 296 K
β = 93.659 (3)°	Prism, colorless
*V* = 1515.54 (10) Å^3^	0.68 × 0.63 × 0.60 mm
*Z* = 4	

### Data collection

**Table:**

STOE IPDS 2 diffractometer	3151 independent reflections
Radiation source: fine-focus sealed tube	2494 reflections with *I* > 2σ(*I*)
Graphite monochromator	*R*_int_ = 0.242
rotation method scans	θ_max_ = 26.5°, θ_min_ = 2.0°
Absorption correction: integration (*X-RED32*; Stoe & Cie, 2002)	*h* = −9→9
*T*_min_ = 0.811, *T*_max_ = 0.850	*k* = −12→12
11192 measured reflections	*l* = −26→22

### Refinement

**Table:**

Refinement on *F*^2^	Primary atom site location: structure-invariant direct methods
Least-squares matrix: full	Secondary atom site location: difference Fourier map
*R*[*F*^2^ > 2σ(*F*^2^)] = 0.036	Hydrogen site location: inferred from neighbouring sites
*wR*(*F*^2^) = 0.110	H-atom parameters constrained
*S* = 1.03	*w* = 1/[σ^2^(*F*_o_^2^) + (0.0708*P*)^2^ + 0.085*P*] where *P* = (*F*_o_^2^ + 2*F*_c_^2^)/3
3151 reflections	(Δ/σ)_max_ < 0.001
208 parameters	Δρ_max_ = 0.26 e Å^−^^3^
0 restraints	Δρ_min_ = −0.23 e Å^−^^3^

### Special details

**Table:**

Geometry. All s.u.'s (except the s.u. in the dihedral angle between two l.s. planes) are estimated using the full covariance matrix. The cell s.u.'s are taken into account individually in the estimation of s.u.'s in distances, angles and torsion angles; correlations between s.u.'s in cell parameters are only used when they are defined by crystal symmetry. An approximate (isotropic) treatment of cell s.u.'s is used for estimating s.u.'s involving l.s. planes.
Refinement. Refinement of *F*^2^ against ALL reflections. The weighted *R*-factor *wR* and goodness of fit *S* are based on *F*^2^, conventional *R*-factors *R* are based on *F*, with *F* set to zero for negative *F*^2^. The threshold expression of *F*^2^ > 2σ(*F*^2^) is used only for calculating *R*-factors(gt) *etc*. and is not relevant to the choice of reflections for refinement. *R*-factors based on *F*^2^ are statistically about twice as large as those based on *F*, and *R*- factors based on ALL data will be even larger.

### Fractional atomic coordinates and isotropic or equivalent isotropic displacement parameters (Å^2^)

**Table:**

	*x*	*y*	*z*	*U*_iso_*/*U*_eq_	
C1	0.1690 (2)	0.43022 (19)	0.57226 (9)	0.0532 (4)	
C2	0.2809 (3)	0.5425 (2)	0.57988 (11)	0.0647 (5)	
H2	0.3807	0.5492	0.5558	0.078*	
C3	0.2448 (3)	0.6445 (2)	0.62308 (11)	0.0638 (5)	
H3	0.3168	0.7220	0.6277	0.077*	
C4	0.0984 (2)	0.62852 (18)	0.65935 (9)	0.0530 (4)	
C5	−0.0099 (3)	0.5154 (2)	0.65435 (10)	0.0627 (5)	
H5	−0.1046	0.5061	0.6807	0.075*	
C6	0.0234 (3)	0.4159 (2)	0.60974 (11)	0.0619 (5)	
H6	−0.0507	0.3396	0.6047	0.074*	
C7	0.2824 (2)	0.1007 (2)	0.60045 (9)	0.0551 (4)	
H7A	0.2072	0.0270	0.5829	0.066*	
H7B	0.2176	0.1526	0.6312	0.066*	
C8	0.4526 (2)	0.04597 (17)	0.63097 (8)	0.0462 (4)	
C9	0.4822 (3)	0.0021 (2)	0.70034 (10)	0.0629 (5)	
H9	0.4132	0.0246	0.7344	0.075*	
C10	0.6266 (3)	−0.0750 (2)	0.70250 (11)	0.0708 (6)	
H10	0.6812	−0.1173	0.7387	0.085*	
C11	0.6856 (3)	−0.0808 (2)	0.63474 (10)	0.0631 (5)	
H11	0.7582	−0.1609	0.6251	0.076*	
C12	0.7690 (2)	0.0595 (2)	0.61967 (10)	0.0578 (5)	
H12A	0.8537	0.0891	0.6541	0.069*	
H12B	0.8269	0.0576	0.5792	0.069*	
C13	0.6017 (2)	0.15046 (18)	0.61523 (8)	0.0470 (4)	
C14	0.5380 (2)	0.2031 (2)	0.54972 (9)	0.0566 (5)	
H14A	0.5733	0.2980	0.5442	0.068*	
H14B	0.5856	0.1480	0.5159	0.068*	
N1	0.0537 (2)	0.73809 (18)	0.70409 (9)	0.0641 (4)	
N2	0.3418 (2)	0.19055 (17)	0.54859 (7)	0.0551 (4)	
O1	0.1109 (2)	0.85355 (16)	0.69498 (9)	0.0860 (5)	
O2	−0.0412 (3)	0.70905 (18)	0.74790 (9)	0.0837 (5)	
O3	0.0442 (2)	0.23390 (18)	0.49970 (8)	0.0769 (4)	
O4	0.3033 (2)	0.36571 (18)	0.46492 (7)	0.0810 (5)	
O5	0.51741 (17)	−0.07105 (13)	0.59750 (7)	0.0592 (3)	
S1	0.20917 (7)	0.30168 (5)	0.51441 (2)	0.06001 (17)	
Cl1	0.60735 (7)	0.28908 (5)	0.67199 (3)	0.06988 (18)	

### Atomic displacement parameters (Å^2^)

**Table:**

	*U*^11^	*U*^22^	*U*^33^	*U*^12^	*U*^13^	*U*^23^
C1	0.0534 (10)	0.0544 (10)	0.0520 (9)	0.0054 (8)	0.0040 (8)	0.0121 (8)
C2	0.0608 (11)	0.0608 (11)	0.0743 (13)	−0.0031 (9)	0.0195 (10)	0.0156 (10)
C3	0.0578 (11)	0.0519 (10)	0.0817 (13)	−0.0070 (9)	0.0052 (10)	0.0105 (10)
C4	0.0515 (10)	0.0492 (9)	0.0571 (10)	0.0048 (8)	−0.0047 (8)	0.0084 (8)
C5	0.0535 (10)	0.0635 (11)	0.0726 (12)	−0.0029 (9)	0.0158 (9)	0.0020 (10)
C6	0.0525 (10)	0.0574 (10)	0.0769 (13)	−0.0088 (9)	0.0119 (9)	−0.0017 (9)
C7	0.0448 (9)	0.0584 (10)	0.0620 (11)	−0.0040 (8)	0.0040 (8)	0.0106 (8)
C8	0.0424 (8)	0.0477 (8)	0.0487 (9)	−0.0034 (7)	0.0035 (7)	0.0019 (7)
C9	0.0616 (12)	0.0735 (12)	0.0541 (10)	−0.0033 (10)	0.0082 (9)	0.0153 (9)
C10	0.0661 (13)	0.0782 (14)	0.0667 (12)	0.0053 (11)	−0.0059 (10)	0.0215 (11)
C11	0.0549 (11)	0.0626 (11)	0.0704 (12)	0.0109 (9)	−0.0069 (9)	−0.0058 (9)
C12	0.0417 (9)	0.0758 (12)	0.0559 (10)	0.0016 (9)	0.0027 (7)	−0.0089 (9)
C13	0.0434 (9)	0.0529 (9)	0.0452 (8)	−0.0062 (7)	0.0068 (7)	−0.0058 (7)
C14	0.0493 (10)	0.0691 (11)	0.0523 (10)	−0.0022 (9)	0.0104 (8)	0.0087 (8)
N1	0.0616 (10)	0.0597 (10)	0.0688 (11)	0.0112 (8)	−0.0126 (8)	−0.0010 (8)
N2	0.0484 (8)	0.0640 (9)	0.0531 (8)	0.0057 (7)	0.0055 (7)	0.0119 (7)
O1	0.0916 (12)	0.0555 (9)	0.1090 (13)	0.0002 (8)	−0.0077 (10)	−0.0062 (9)
O2	0.0914 (12)	0.0848 (11)	0.0759 (10)	0.0132 (9)	0.0141 (9)	−0.0065 (8)
O3	0.0687 (10)	0.0855 (10)	0.0735 (9)	0.0134 (8)	−0.0203 (7)	−0.0094 (8)
O4	0.1031 (13)	0.0910 (11)	0.0507 (8)	0.0235 (10)	0.0181 (8)	0.0212 (8)
O5	0.0568 (8)	0.0521 (7)	0.0672 (8)	0.0010 (6)	−0.0074 (6)	−0.0104 (6)
S1	0.0645 (3)	0.0674 (3)	0.0475 (3)	0.0122 (2)	−0.0015 (2)	0.0068 (2)
Cl1	0.0700 (3)	0.0652 (3)	0.0748 (3)	−0.0109 (2)	0.0081 (3)	−0.0243 (2)

### Geometric parameters (Å, º)

**Table:**

C1—C2	1.382 (3)	C9—H9	0.9300
C1—C6	1.390 (3)	C10—C11	1.503 (3)
C1—S1	1.773 (2)	C10—H10	0.9300
C2—C3	1.376 (3)	C11—O5	1.443 (2)
C2—H2	0.9300	C11—C12	1.542 (3)
C3—C4	1.381 (3)	C11—H11	0.9800
C3—H3	0.9300	C12—C13	1.536 (3)
C4—C5	1.369 (3)	C12—H12A	0.9700
C4—N1	1.467 (3)	C12—H12B	0.9700
C5—C6	1.374 (3)	C13—C14	1.503 (3)
C5—H5	0.9300	C13—Cl1	1.7896 (17)
C6—H6	0.9300	C14—N2	1.479 (2)
C7—N2	1.478 (2)	C14—H14A	0.9700
C7—C8	1.490 (3)	C14—H14B	0.9700
C7—H7A	0.9700	N1—O1	1.222 (2)
C7—H7B	0.9700	N1—O2	1.225 (2)
C8—O5	1.435 (2)	N2—S1	1.6050 (16)
C8—C9	1.505 (3)	O3—S1	1.4208 (17)
C8—C13	1.563 (2)	O4—S1	1.4272 (15)
C9—C10	1.318 (3)		
			
C2—C1—C6	120.70 (19)	O5—C11—C12	100.80 (15)
C2—C1—S1	120.42 (14)	C10—C11—C12	107.76 (17)
C6—C1—S1	118.88 (15)	O5—C11—H11	115.0
C3—C2—C1	120.03 (18)	C10—C11—H11	115.0
C3—C2—H2	120.0	C12—C11—H11	115.0
C1—C2—H2	120.0	C13—C12—C11	100.37 (14)
C2—C3—C4	118.14 (19)	C13—C12—H12A	111.7
C2—C3—H3	120.9	C11—C12—H12A	111.7
C4—C3—H3	120.9	C13—C12—H12B	111.7
C5—C4—C3	122.68 (18)	C11—C12—H12B	111.7
C5—C4—N1	118.22 (17)	H12A—C12—H12B	109.5
C3—C4—N1	119.08 (18)	C14—C13—C12	117.59 (14)
C4—C5—C6	118.95 (17)	C14—C13—C8	102.66 (14)
C4—C5—H5	120.5	C12—C13—C8	102.01 (14)
C6—C5—H5	120.5	C14—C13—Cl1	109.40 (13)
C5—C6—C1	119.42 (18)	C12—C13—Cl1	114.20 (13)
C5—C6—H6	120.3	C8—C13—Cl1	109.84 (11)
C1—C6—H6	120.3	N2—C14—C13	104.26 (13)
N2—C7—C8	103.29 (13)	N2—C14—H14A	110.9
N2—C7—H7A	111.1	C13—C14—H14A	110.9
C8—C7—H7A	111.1	N2—C14—H14B	110.9
N2—C7—H7B	111.1	C13—C14—H14B	110.9
C8—C7—H7B	111.1	H14A—C14—H14B	108.9
H7A—C7—H7B	109.1	O1—N1—O2	123.69 (19)
O5—C8—C7	112.73 (15)	O1—N1—C4	118.23 (19)
O5—C8—C9	101.80 (14)	O2—N1—C4	118.07 (18)
C7—C8—C9	125.36 (15)	C7—N2—C14	112.62 (14)
O5—C8—C13	98.31 (12)	C7—N2—S1	120.83 (12)
C7—C8—C13	106.67 (14)	C14—N2—S1	122.85 (13)
C9—C8—C13	108.70 (15)	C8—O5—C11	96.05 (13)
C10—C9—C8	105.36 (17)	O3—S1—O4	120.93 (11)
C10—C9—H9	127.3	O3—S1—N2	106.96 (10)
C8—C9—H9	127.3	O4—S1—N2	106.85 (9)
C9—C10—C11	106.33 (18)	O3—S1—C1	106.78 (10)
C9—C10—H10	126.8	O4—S1—C1	107.07 (10)
C11—C10—H10	126.8	N2—S1—C1	107.66 (9)
O5—C11—C10	101.43 (16)		
			
C6—C1—C2—C3	−2.6 (3)	C7—C8—C13—Cl1	83.44 (16)
S1—C1—C2—C3	177.44 (17)	C9—C8—C13—Cl1	−54.21 (17)
C1—C2—C3—C4	2.0 (3)	C12—C13—C14—N2	139.34 (16)
C2—C3—C4—C5	0.6 (3)	C8—C13—C14—N2	28.35 (18)
C2—C3—C4—N1	−177.90 (18)	Cl1—C13—C14—N2	−88.26 (15)
C3—C4—C5—C6	−2.6 (3)	C5—C4—N1—O1	−157.46 (19)
N1—C4—C5—C6	175.92 (19)	C3—C4—N1—O1	21.1 (3)
C4—C5—C6—C1	1.9 (3)	C5—C4—N1—O2	21.4 (3)
C2—C1—C6—C5	0.6 (3)	C3—C4—N1—O2	−159.99 (19)
S1—C1—C6—C5	−179.46 (16)	C8—C7—N2—C14	−5.1 (2)
N2—C7—C8—O5	−83.60 (17)	C8—C7—N2—S1	−164.04 (13)
N2—C7—C8—C9	151.72 (18)	C13—C14—N2—C7	−15.5 (2)
N2—C7—C8—C13	23.20 (18)	C13—C14—N2—S1	142.87 (14)
O5—C8—C9—C10	32.8 (2)	C7—C8—O5—C11	173.09 (15)
C7—C8—C9—C10	162.04 (19)	C9—C8—O5—C11	−50.15 (16)
C13—C8—C9—C10	−70.3 (2)	C13—C8—O5—C11	61.04 (15)
C8—C9—C10—C11	−0.7 (2)	C10—C11—O5—C8	49.46 (17)
C9—C10—C11—O5	−31.3 (2)	C12—C11—O5—C8	−61.35 (16)
C9—C10—C11—C12	74.1 (2)	C7—N2—S1—O3	−46.28 (18)
O5—C11—C12—C13	35.15 (17)	C14—N2—S1—O3	157.01 (15)
C10—C11—C12—C13	−70.69 (18)	C7—N2—S1—O4	−177.11 (15)
C11—C12—C13—C14	−109.52 (18)	C14—N2—S1—O4	26.18 (18)
C11—C12—C13—C8	1.83 (17)	C7—N2—S1—C1	68.16 (17)
C11—C12—C13—Cl1	120.25 (14)	C14—N2—S1—C1	−88.54 (16)
O5—C8—C13—C14	83.97 (15)	C2—C1—S1—O3	−157.40 (17)
C7—C8—C13—C14	−32.86 (17)	C6—C1—S1—O3	22.68 (19)
C9—C8—C13—C14	−170.51 (15)	C2—C1—S1—O4	−26.55 (19)
O5—C8—C13—C12	−38.24 (15)	C6—C1—S1—O4	153.53 (16)
C7—C8—C13—C12	−155.07 (15)	C2—C1—S1—N2	88.03 (17)
C9—C8—C13—C12	67.28 (17)	C6—C1—S1—N2	−91.89 (17)
O5—C8—C13—Cl1	−159.73 (11)		

### Hydrogen-bond geometry (Å, º)

**Table:**

*D*—H···*A*	*D*—H	H···*A*	*D*···*A*	*D*—H···*A*
C2—H2···O4^i^	0.93	2.58	3.437 (3)	154
C14—H14*B*···O5^ii^	0.97	2.54	3.317 (2)	137

Symmetry codes: (i) −*x*+1, −*y*+1, −*z*+1; (ii) −*x*+1, −*y*, −*z*+1.
